# Parkin mediates the mitochondrial dysfunction through mRpL18

**DOI:** 10.1016/j.jbc.2025.110208

**Published:** 2025-05-08

**Authors:** Xiuxiu Ti, Hui Zuo, Guochun Zhao, Yuwei Li, Minghui Du, Liwen Xu, Shengnan Li, Zhaoliang Shan, Yuxue Gao, Guangming Gan, Yan Wang, Qing Zhang

**Affiliations:** 1 State Key Laboratory of Pharmaceutical Biotechnology and MOE Key Laboratory of Model Animals for Disease Study, Jiangsu Key Laboratory of Molecular Medicine, Model Animal Research Center, School of Medicine, Nanjing University, Nanjing, China; 2 Department of Cardiovascular Medicine, Beijing Hospital, National Center of Gerontology, Institute of Geriatric Medicine, Chinese Academy of Medical Sciences, Beijing, China; 3 Key Laboratory of Developmental Genes and Human Disease, School of Life Science and Technology, School of Medicine, Southeast University, Nanjing, China

**Keywords:** Parkinson's disease, Parkin, mRpL18, Drp1

## Abstract

Loss of function of *parkin* leads to mitochondrial dysfunction, which is closely related to Parkinson's disease. However, the *in vivo* mechanism is far from clear. One dogma is that impaired Parkin causes dysfunction of mitophagy mediated by Pink1-Parkin axis. The other is that impaired Parkin causes Mfn accumulation which leads to mitochondrial dysfunction. Surprisingly, in *Drosophila* muscles, the first dogma is not applicable; for the second dogma, our study suggests that Parkin mediates mitochondrial dysfunction through the synergy of both Marf and mitochondrial protein mRpL18 got from our genome-wide screen, whose RNAi rescues *parkin* RNAi phenotype. Mechanistically, we found that impaired Parkin upregulated both transcription and protein levels of mRpL18 dependent on its E3 ligase activity, causing mRpL18 accumulation outside mitochondria. Consequently, cytosolic-accumulated mRpL18 competitively bound Drp1, leading to the reduction of the binding of Drp1 to its receptor Fis1, which finally inhibited mitochondrial fission and tipped the balance to mitochondrial hyperfusion, thereby affected the mitochondrial function. Taken together, our study suggests that impaired Parkin causes mitochondrial hyperfusion due to two reasons: (1) Parkin defect impairs Pink1-Parkin axis-mediated Marf degradation, which promotes mitochondrial fusion; (2) Parkin defect causes mRpL18 accumulation, which inhibits Drp1/Fis1-mediated mitochondrial fission. These two ways together drive Parkin-mediated mitochondrial hyperfusion. Therefore, knockdown of either *marf* or *mRpL18* can prevent mitochondrial hyperfusion, leading to the rescue of Parkin defect-triggered fly wing phenotypes. Overall, our study unveils a new facet of how Parkin regulates mitochondrial morphology, which provides new insights for the understanding and treatment of Parkinson's disease.

Parkinson’s disease (PD) is the second prevalent neurodegenerative movement disorder which affects about 2% of the population above the age of 60, causing both motor and non-motor symptoms (1, 2). The cardinal motor symptoms include rest tremors, rigidity, bradykinesia (slowed movement), and postural instability, whereas nonmotor symptoms include abnormalities of mood, cognition, sleep, and autonomic function (3). Although PD is characterized by a pathophysiologic loss or degeneration of dopaminergic neurons in the substantia nigra of the midbrain and accumulation of misfolded α-synuclein in intra-cytoplasmic inclusions called Lewy bodies (4, 5), the etiology of PD in most patients is still unknown.

A lot of risk factors which impair mitochondrial functions, including familial gene mutations, aging, and environmental chemicals exposure (*e.g.*, pesticide and synthetic heroin) have been reported to be closely associated with PD, indicating that the mitochondrial dysfunction may be the underlying reason for many cases of PD (1, 6, 7, 8, 9, 10, 11, 12). Subsequent studies have verified that mitochondria function as a central mechanism in multiple neurodegenerative diseases including PD (13). Therefore, based on these studies, mitochondria have now been explored as a therapeutic target of kinds of neurodegenerative diseases, such as PD and AD (14, 15, 16).

Although most cases with PD are sporadic, more than 20 responsible genes in familial cases were identified since 1997, including autosomal dominant and recessive genes, such as *snca*, *lrrk2*, *vps35*, *pink1*, *parkin*, *dj-1*, *dnajc6*, *fbxo7*, *synj1*, *atp13a2*, and *pla2g6* (7, 17, 18). Among them, some variants of autosomal recessive inheritance are present in early onset Parkinson’s disease. Approximately 50% of early-onset forms of PD have been linked to bi-allelic mutations of *parkin*, *pink1*, and *dj-1* (19, 20, 21, 22). Each of them is implicated in maintaining proper mitochondrial function, which is particularly important for neuronal health.

*parkin* encodes an E3 ligase which together with kinase Pink1 acts cooperatively in sensing mitochondrial functional state and marking damaged mitochondria for disposal through mitophagy, thus fine-tuning mitochondrial network and preserving energy metabolism (23, 24, 25, 26, 27). In addition to affecting mitophagy, Parkin also modulates mitochondrial morphology by dynamically controlling mitochondrial fission and fusion (28). In *Drosophila*, loss-of-function mutants of *parkin* exhibit shortened lifespan, motor deficits, and other phenotypes, such as excessive mitochondrial fusion (29). Of note, it is different from *Drosophila*, in mammalian system, loss of function of *parkin* shows either mitochondrial hyperfission or hyperfusion phenotypes dependent on different cell types (30, 31, 32, 33). In vigorously dividing mammalian cells, it tends to show mitochondrial hyperfission phenotypes (31), while in nondividing cells such as rat midbrain dopaminergic neurons, similar to *Drosophila*, dysfunction of *parkin* tends to show mitochondrial hyperfusion phenotypes (33). Consistently, mitochondrial hyperfusion is also presented in dopamine neurons of *parkin*-mutated patients with PD (34, 35). Overall, loss of function of *parkin* in *Drosophila* and mammalian dopaminergic neurons show similar mitochondrial hyperfusion phenotypes, indicating that we can use *Drosophila* model to study how loss of *parkin* function affects mitochondrial function, which is helpful to understand the etiology of Parkin-mediated PD.

As mentioned earlier, we have known some about how *parkin* mutations lead to mitochondrial dysfunction (26, 29, 36, 37, 38, 39), but the *in vivo* mechanism is far from clear. One of the dogmas is that impaired Parkin causes dysfunction of mitophagy mediated by Pink1-Parkin axis (23). The other is that impaired Parkin causes Mfn accumulation which leads to mitochondrial dysfunction. Surprisingly, in *Drosophila* muscles, as reported, the first dogma does not apply (40); for the second dogma, our study suggests that Parkin mediates the mitochondrial dysfunction in a synergistic manner, mediated by Marf (28, 29, 41), and the mitochondrial protein mRpL18, which was obtained in our whole-genome screen, and its RNAi rescued the *parkin* RNAi phenotype. The results support the idea that there are two reasons why impaired Parkin causes mitochondrial hyperfusion: (1) Parkin defect impairs Pink1-Parkin axis-mediated Marf degradation, which promotes mitochondrial fusion; (2) impaired Parkin causes mRpL18 accumulation, which inhibits Drp1/Fis1-mediated mitochondrial fission. Together, these two ways function together to drive Parkin-mediated mitochondrial hyperfusion. Therefore, knockdown of either *marf* or *mRpL18* can prevent mitochondrial hyperfusion, thus rescuing Parkin deficiency-triggered fly wing phenotypes. Overall, our study unveils a new facet of how Parkin regulates the mitochondrial morphology, which provides new insights for the understanding and treatment of Parkinson's disease.

## Results

### Establishing a *parkin* RNAi-mediated *Drosophila* mitochondrial defect model

**Figure 1:**
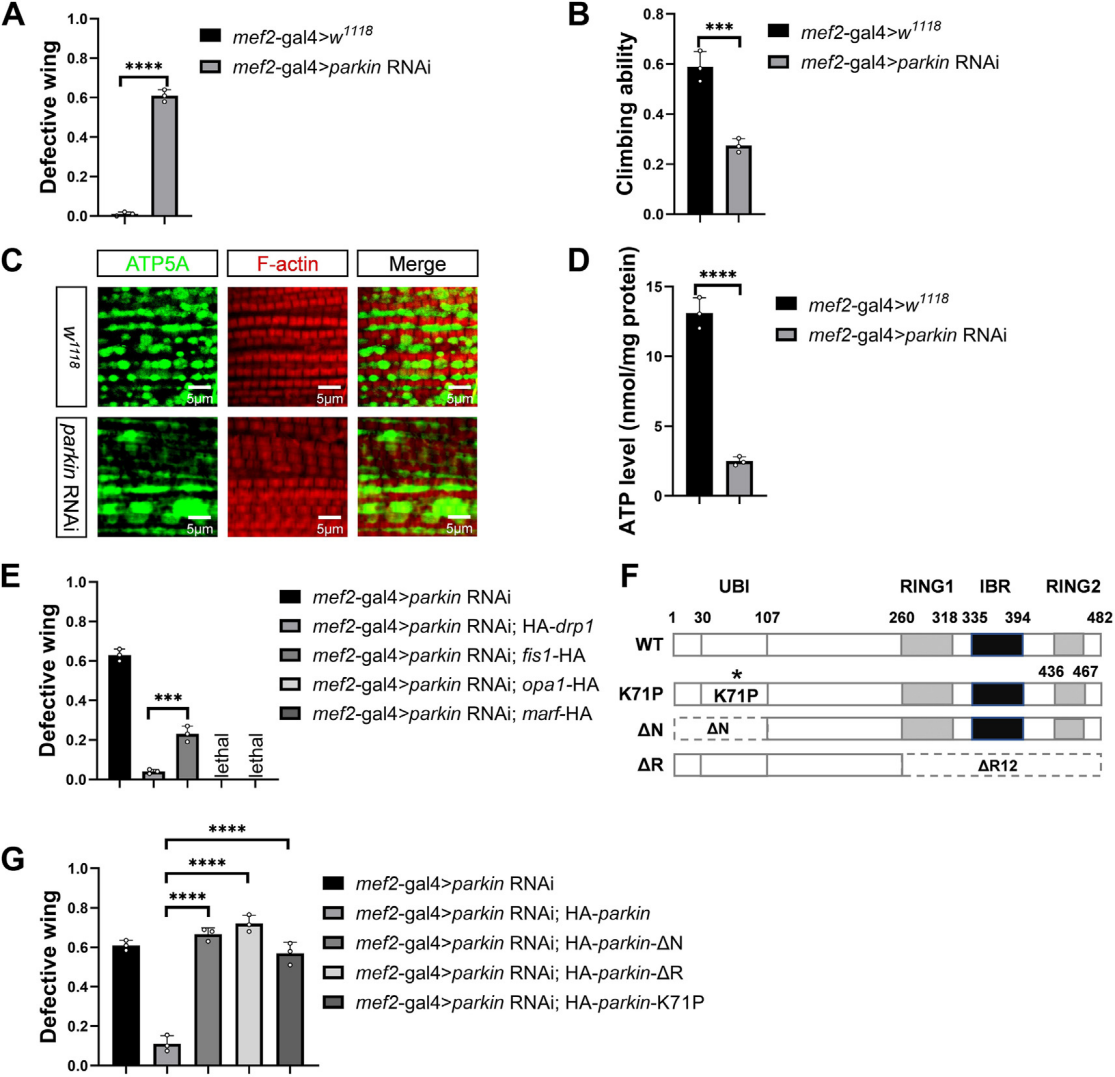
**Establishing a *parkin* RNAi-mediated *Drosophila* mitochondrial defect model.***A*, knockdown of *parkin* in *Drosophila* muscles produced abnormal wing phenotypes. *B*, *parkin* RNAi flies exhibited impaired locomotion. *C*, *parkin* RNAi flies exhibited hyperfused mitochondrial morphology. *D*, *parkin* RNAi flies showed a significantly decreased ATP level. *E*, overexpression of Drp1 rescued *parkin* RNAi-mediated abnormal wing phenotypes. *F*, schematic diagram of the structures of wild-type and E3 ligase-dead Parkin. *G*, wild-type Parkin could, but its E3 ligase-dead mutants could not rescue the *parkin* RNAi-mediated abnormal wing phenotypes. Values in bar graphs represented mean ± SD of three biologically independent experiments. In each biologically independent experiment, n = 200 for the analysis of defective wing; n = 100 for the analysis of climbing ability. For the analysis of mitochondrial morphology, the representative image for every sample was shown from six flies. For *A*, *B*, *D* and *E*, two tailed unpaired *t* test was used. ∗ *p*-value < 0.05; ∗∗ *p*-value < 0.01; ∗∗∗ *p*-value < 0.001; ∗∗∗∗ *p*-value < 0.0001. For *G*, one-way ANOVA was used, (F) (4, 10) = 108.5, *p*-value < 0.0001. Tukey’s multiple comparisons tests were used for the *post hoc* tests.

Surprisingly, until now, genome-wide RNAi screen to identify the rescue genes of *parkin* RNAi phenotype has not been conducted. To find the potential *parkin* RNAi-phenotype mediators, we started to establish a *parkin* RNAi-mediated mitochondrial defect *Drosophila* model. In *Drosophila* muscles, knockdown of *parkin* by a muscle specific *mef2*-gal4 driving UAS*-parkin* RNAi expression caused apparent erect or dropped wing phenotypes (Fig. 1*A*). Except for that, *parkin* RNAi flies also exhibited impaired locomotion and mitochondrial hyperfusion morphology (Fig. 1*B*, *C*). Since mitochondria produce the majority of cellular energy ATP, we next tested ATP level and used it to demarcate mitochondrial function. Compared with controls, *parkin* RNAi flies showed a significant decreased ATP level, indicating that knockdown of *parkin* indeed impairs mitochondrial function (Fig. 1*D*). To explore the relationship among the phenotypes, we overexpressed *drp1* to inhibit the hyperfused mitochondrial morphology (37, 41, 42, 43, 44) and found that *parkin* RNAi-mediated abnormal wing phenotypes were rescued (Fig. 1*E*), suggesting that knockdown of *parkin* results in change of mitochondrial morphology, which causes abnormal wing postures.

Parkin is an E3 ligase with Ubl, RING1, IBR, and RING2 domains, which are closely related to its ligase activity (38, 45, 46). To further address whether Parkin-mediating mitochondrial defect is dependent on its E3 ligase activity, we made several Parkin E3 ligase-dead mutants including *parkin-ΔN*, *parkin-ΔR*, and *parkin-K71P*. In detail, Parkin-ΔN and Parkin-ΔR were made by removing the amino acids from Position 1 to 107 (Ubl domain) and 260 to 482 (RING1 + IBR + RING2 domain), respectively. Parkin-K71P was made by mutation of the corresponding amino acid based on human Parkinson's pathogenic mutant Parkin-R42P (47), leading to the destruction of Ubl structure, thus preventing the interaction between Parkin and substrate protein (Fig. 1*F*). Applying *parkin-ΔN*, *parkin-ΔR*, and *parkin-K71P* transgenic flies, we found that unlike the WT Parkin, these ligase-dead mutants failed to restore the *parkin* RNAi-mediated abnormal wing phenotypes (Fig. 1*G*), suggesting that Parkin rescues abnormal wing phenotypes dependent on its E3 ligase activity. Taken together, we had generated a mitochondrial defect fly model by knockdown of *parkin* with its RNAi, which had visible wing phenotypes and was easy for subsequent genetic screens.

### The genetic screen demonstrates that mRpL18 mediates the *parkin* RNAi-triggered mitochondrial defect effects

**Figure 2:**
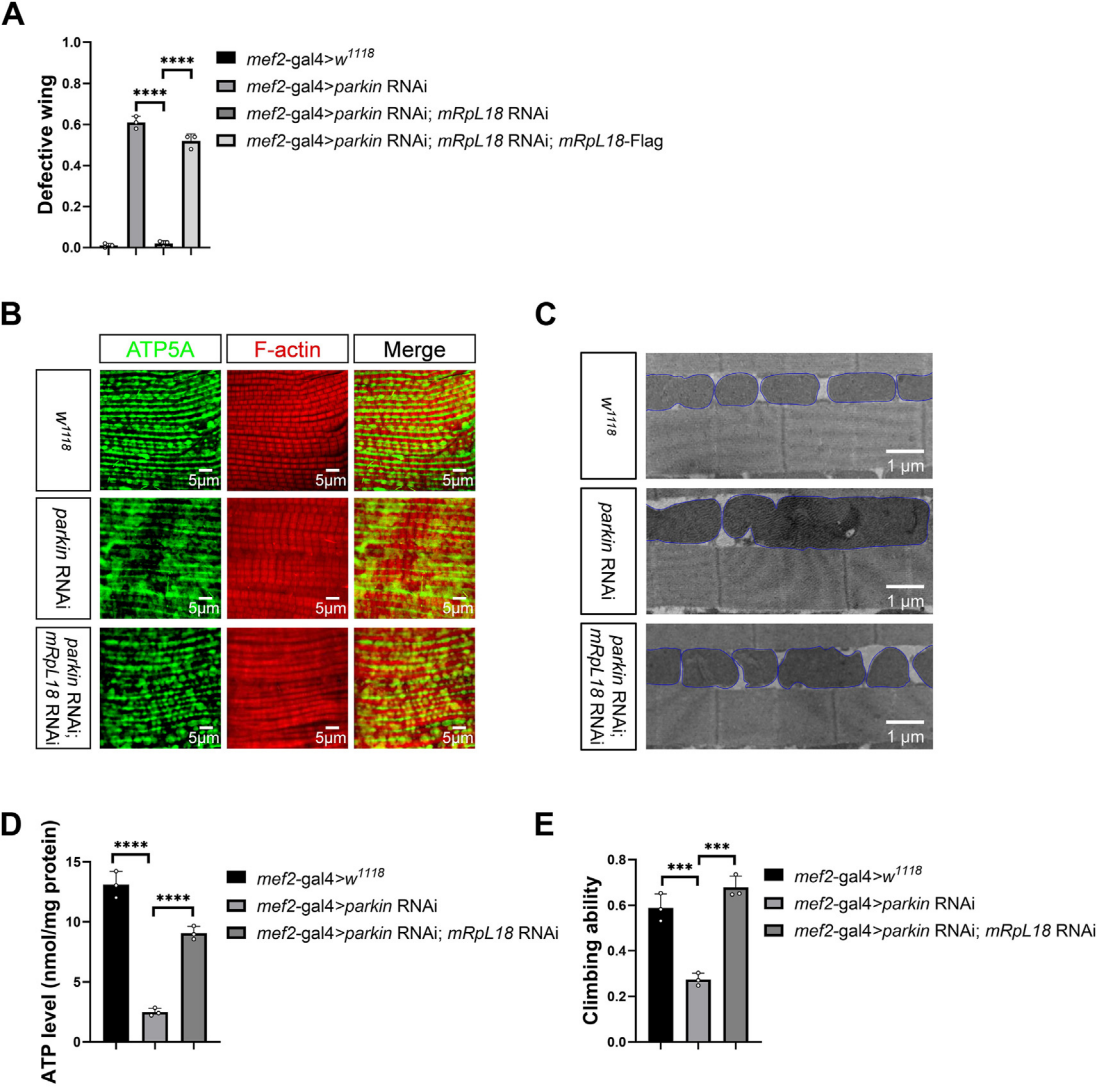
**The genetic screen demonstrates that mRpL18 mediates the *parkin* RNAi-triggered mitochondrial defect effects.***A-E*, knockdown of *mRpL18* could rescue *parkin* RNAi-mediated abnormal wing phenotypes (*A*), mitochondrial hyperfusion (*B* and *C*), decreased ATP level (*D*), and decreased climbing ability (*E*). The mitochondria boundaries were marked with blue lines in (*C*). Values in bar graphs represented mean ± SD of three biologically independent experiments. In each biologically independent experiment, n = 200 for analysis of defective wings; n = 100 for analysis of climbing ability. For the confocal or TEM analysis of mitochondrial morphology, the representative image for every sample was shown from six flies. One-way ANOVA was used for *A*, *D,* and *E*, their F and *p*-values were F (3, 8) = 516.4, *p*-value < 0.0001; F (2, 6) = 158.8, *p*-value < 0.0001 and F (2, 6) = 58.75, *p*-value = 0.0001, respectively. The *post hoc* test results following one-way ANOVA were indicated by significant difference star(s), ∗ *p*-value < 0.05; ∗∗ *p*-value < 0.01; ∗∗∗ *p*-value < 0.001; ∗∗∗∗ *p*-value < 0.0001.

Using the built model (UAS-*parkin* RNAi; *mef2*-gal4), we performed RNAi screen among approximately 7000 conserved genes between flies and mammals to check that knockdown of which genes can rescue the dropped and erected wing phenotypes of *parkin* RNAi. After the screen, we surprisingly found that knockdown of the nuclear-encoded mitochondrial ribosomal protein L18 (*mRpL18*) gene rescued *parkin* RNAi-mediated abnormal wing phenotypes (Fig. 2*A*). Consistently, overexpressing *mRpL18* reversed the rescue effect of *mRpL18* RNAi (Fig. 2*A*), indicating that no off-target effect occurs and mRpL18 indeed mediates the *parkin* RNAi-triggered mitochondrial defect effects.

Given *parkin* RNAi-mediated wing phenotypes were mediated by excessive mitochondrial fusion, we next tested whether knockdown of *mRpL18* rescues the phenotypes by modulating mitochondrial morphology. Labeling mitochondria of fly thoracic muscles with ATP5A antibody, we found that knockdown of *mRpL18* rescued *parkin* RNAi-mediated excessive mitochondrial fusion (Fig. 2*B*). Consistently, the transmission electron microscopy (TEM) images also showed the similar results (Fig. 2*C*). Moreover, knockdown of *mRpL18* also rescued the mitochondrial ATP level (Fig. 2*D*) and exercise capacity (Fig. 2*E*). Together, these and aforementioned *drp1* overexpression results support that knockdown of *mRpL18* rescues the wing phenotypes through modulating mitochondrial morphology.

### The cytosolic mRpL18 mediates the *parkin* RNAi-triggered mitochondrial defect effects

**Figure 3:**
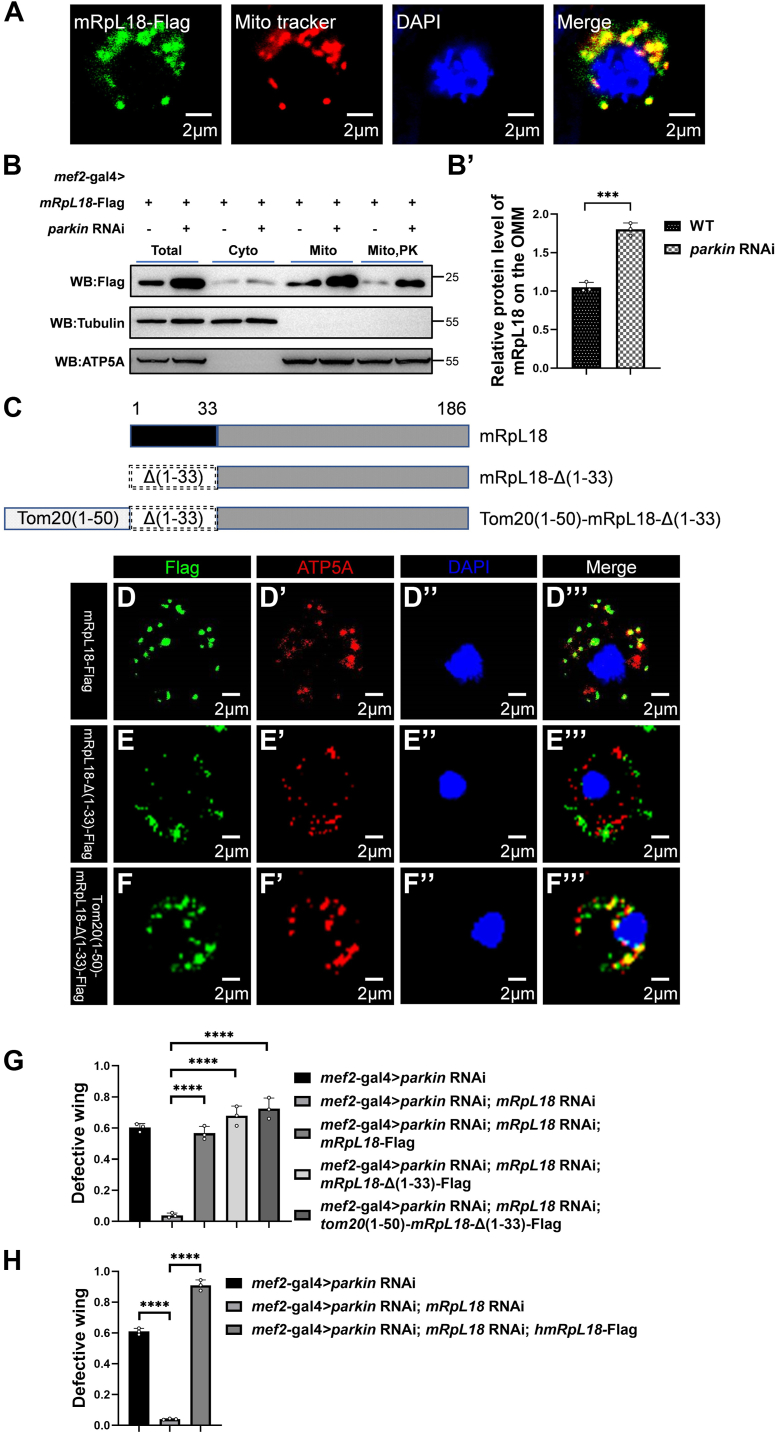
**The cytosolic mRpL18 mediates the *parkin* RNAi-triggered mitochondrial defect effects.***A*, mRpL18 was localized in mitochondria in S2 cells. n = 20. *B–B’*, mRpL18 was mainly accumulated on the cytosolic facing surface of OMM. Western blots were representative of three biologically independent experiments and the bar graph showed the mean ± SD. *C*, schematic diagram of wild-type and mutant mRpL18 structures. *D–F*, cellular localization of wild-type mRpL18 (*D*–*D’’’*), mutant mRpL18-Δ(1–33) (*E*–*E’’’*) and Tom20(1–50)-mRpL18-Δ(1–33) (*F*–*F’’’*). For every sample, n ≥ 20. *G*, knockdown of *mRpL18* could rescue *parkin* RNAi-mediated abnormal wing phenotypes, WT mRpL18 reversed the rescue effects, mutant mRpL18-Δ(1–33) and Tom20(1–50)-mRpL18-Δ(1–33) aggravated *parkin* RNAi-mediated abnormal wing phenotypes. *H*, human homolog of mRpL18 could reverse the rescue effects of *mRpL18* RNAi. In each biologically independent experiment, n = 200 for the analysis of defective wings. For *B’*, two-tailed unpaired *t* test was used. *p*-value = 0.0002. For *G* and *H*, one-way ANOVA was used, their F and *p*-values were F (4, 10) = 102.4, *p*-value < 0.0001 and F (2, 6) = 1094, *p*-value < 0.0001, respectively. The *post hoc* test results following one-way ANOVA were indicated by significant difference star(s), ∗ *p*-value < 0.05; ∗∗ *p*-value < 0.01; ∗∗∗ *p*-value < 0.001; ∗∗∗∗ *p*-value < 0.0001.

Given the knockdown of *mRpL18* rescued the *parkin* RNAi-mediated phenotypes, we next tested whether knockdown of *parkin* affects mRpL18 protein level. mRpL18, as mitochondrial ribosomal protein (48), was localized in mitochondria (Fig. 3*A*). However, *parkin* RNAi might affect it into mitochondrial matrix. As shown in Figure 3*B*, knockdown of *parkin* dramatically upregulated mRpL18 level. After separating cytoplasmic and mitochondrial parts, we found the majority of mRpL18 was distributed on mitochondria. Further using proteinase K to degrade the cytosolic facing proteins on outer mitochondrial membrane (OMM), we found that mRpL18 level was dramatically downregulated, suggesting that mRpL18 was mainly accumulated on the cytosolic facing surface of OMM. Overall, the results indicate that knockdown of *parkin* increases mRpL18 accumulation outside mitochondria.

To address that mRpL18 is located inside or outside the mitochondria to mediate the adverse effects, we made two cytosolic localized forms of mRpL18, named mRpL18-Δ(1–33) and Tom20(1–50)-mRpL18-Δ(1–33), respectively. MitoProtⅡ-v1.101 software analysis indicated the mitochondrial signal peptide of mRpL18 is composed of its N-terminal 1 to 33 amino acids. mRpL18-Δ(1–33), which lacks the mitochondrial signal peptide (1–33aa), was located in the cytosol instead of mitochondrial matrix (Fig. 3*C*, *E–E’’’*). Translocase of outer membrane 20 (Tom20) is an outer mitochondrial membrane protein determined by its N-terminal 1 to 50 amino acids (49). Tom20(1–50)-mRpL18-Δ(1–33) was constructed by linking 1 to 50aa of Tom20 to the N-terminus of mRpL18-Δ(1–33), leading to its anchoring on the mitochondrial outer membrane (Fig. 3*C*, *F–F’’’*). When overexpressing mRpL18-Δ(1–33) and Tom20(1–50)-mRpL18-Δ(1–33) in *mRpL18* RNAi background, we found that they behaved like WT mRpL18 to aggravate *parkin* RNAi-mediated abnormal wing phenotypes (Fig. 3*G*), suggesting the cytosolic mRpL18 is sufficient to mediate mitochondrial adverse effects. Importantly, similar to *Drosophila* mRpL18, its human homolog hmRpL18 also reversed *mRpL18* RNAi-mediated rescue effects (Fig. 3*H*), indicating that the function of hmRpL18 is evolutionarily conserved.

### Parkin affects mRpL18 transcription through transcription factor Ribbon

**Figure 4:**
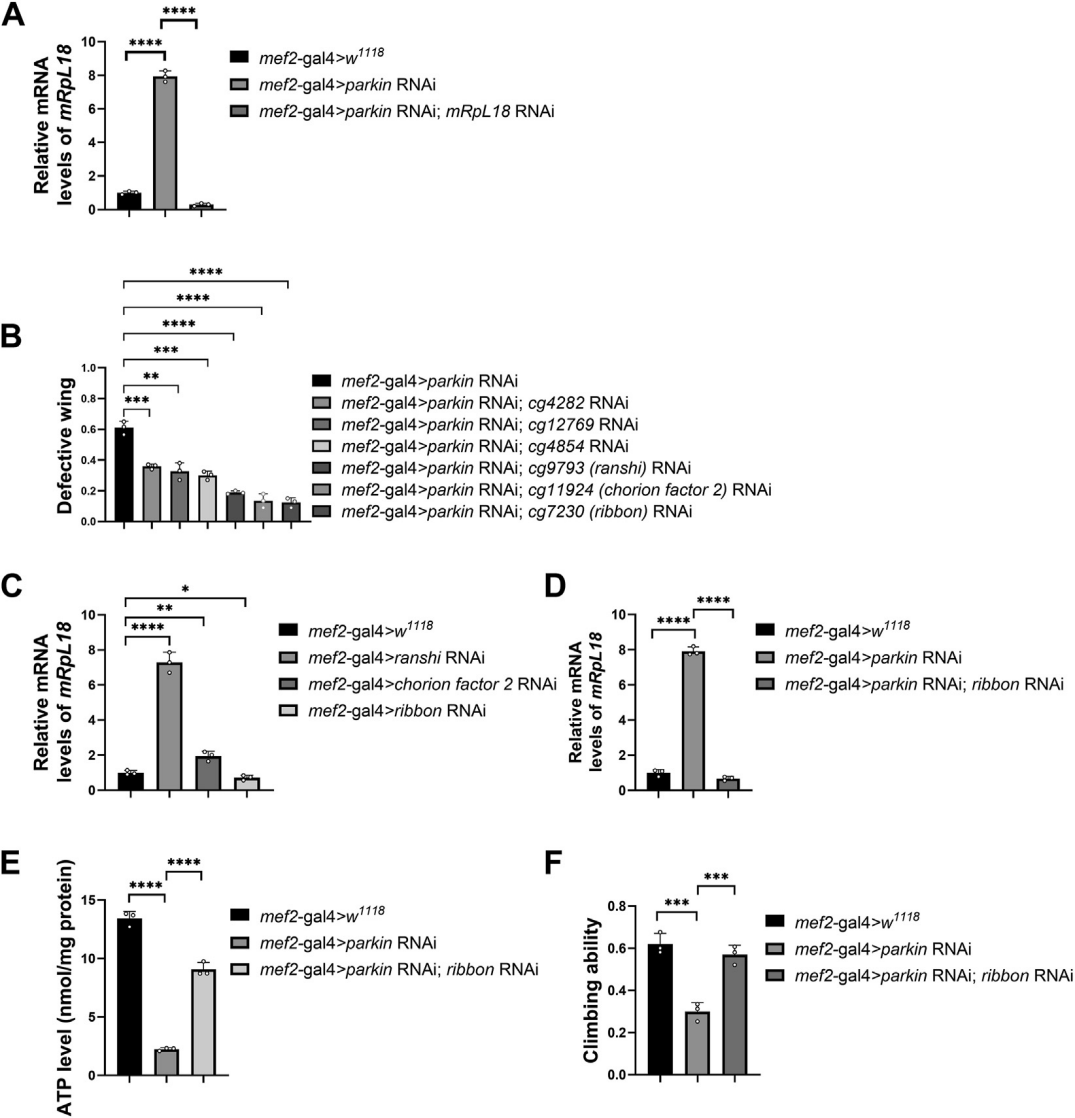
**Parkin affects mRpL18 transcription through transcription factor Ribbon.***A*, knockdown of *parkin* significantly upregulated the mRNA level of *mRpL18*. *B*, knockdown of several transcription factors modulated *parkin* RNAi-mediated wing phenotypes. *C*, knockdown of indicated transcription factors modulated the mRNA level of mRpL18. *D*, knockdown of *ribbon* downregulated the mRNA level of mRpL18 in *parkin* RNAi background. *E*, knockdown of *ribbon* rescued the decreased mitochondrial ATP level caused by *parkin* RNAi. *F*, knockdown of *ribbon* rescued *parkin* RNAi-induced impairment of climbing ability. Values in bar graphs represented mean ± SD of three biologically independent experiments. In each biologically independent experiment, n = 200 for the analysis of defective wings; n = 100 for the analysis of climbing ability. For *A*, one-way ANOVA was used, F (2, 6) = 1256, *p*-value < 0.0001. For *B* and *C*, a two-tailed unpaired *t-test* was used. For *D*, *E,* and *F*, one-way ANOVA was used, their F and *p*-values were F (2, 6) = 1378, *p*-value < 0.0001; F (2, 6) = 381.1, *p*-value < 0.0001, and F (2, 6) = 42.88, *p*-value = 0.0003, respectively. The *post hoc* test results following one-way ANOVA were indicated by significant difference star(s), ∗ *p*-value < 0.05; ∗∗ *p*-value < 0.01; ∗∗∗ *p*-value < 0.001; ∗∗∗∗ *p*-value < 0.0001.

Given that *parkin* affected mRpL18 protein level, we first tested whether Parkin regulates mRpL18 transcription. As shown in Figure 4*A*, the mRNA level of *mRpL18* significantly increased after knockdown of *parkin*, indicating Parkin indeed regulates mRpL18 transcription.

Parkin, as an E3 ligase, does not function as a transcription factor, suggesting that it regulates *parkin* RNAi-initiated mRpL18 transcription through an intermediate transcription factor. Among our screen list, some transcription factors (encoded by *cg4282*, *cg12769 cg4854, cg9793*, *cg11924,* and *cg7230*) showed partial rescue effects (Fig. 4*B*). Among them, *cg9793*, *cg11924,* and *cg7230* showed slightly better rescue effects, named *ranshi*, *chorion factor 2*, and *ribbon*, respectively (50). Then we tested whether these three transcription factors regulate *mRpL18* transcription. As shown in Figure 4*C*, only the knockdown of *ribbon* downregulated the mRNA level of *mRpL18*, suggesting that Ribbon is a candidate transcription factor. Following experiments showed that *ribbon* RNAi also reversed the upregulation of *mRpL18* mRNA level in *parkin* RNAi background (Fig. 4*D*), partially rescued the decreased ATP level (Fig. 4*E*) and impaired locomotion (Fig. 4*F*), further indicating that Parkin modulates the transcription of *mRpL18* through transcription factor Ribbon.

### Parkin modulates Ribbon cellular localization to affect mRpL18 transcription

**Figure 5:**
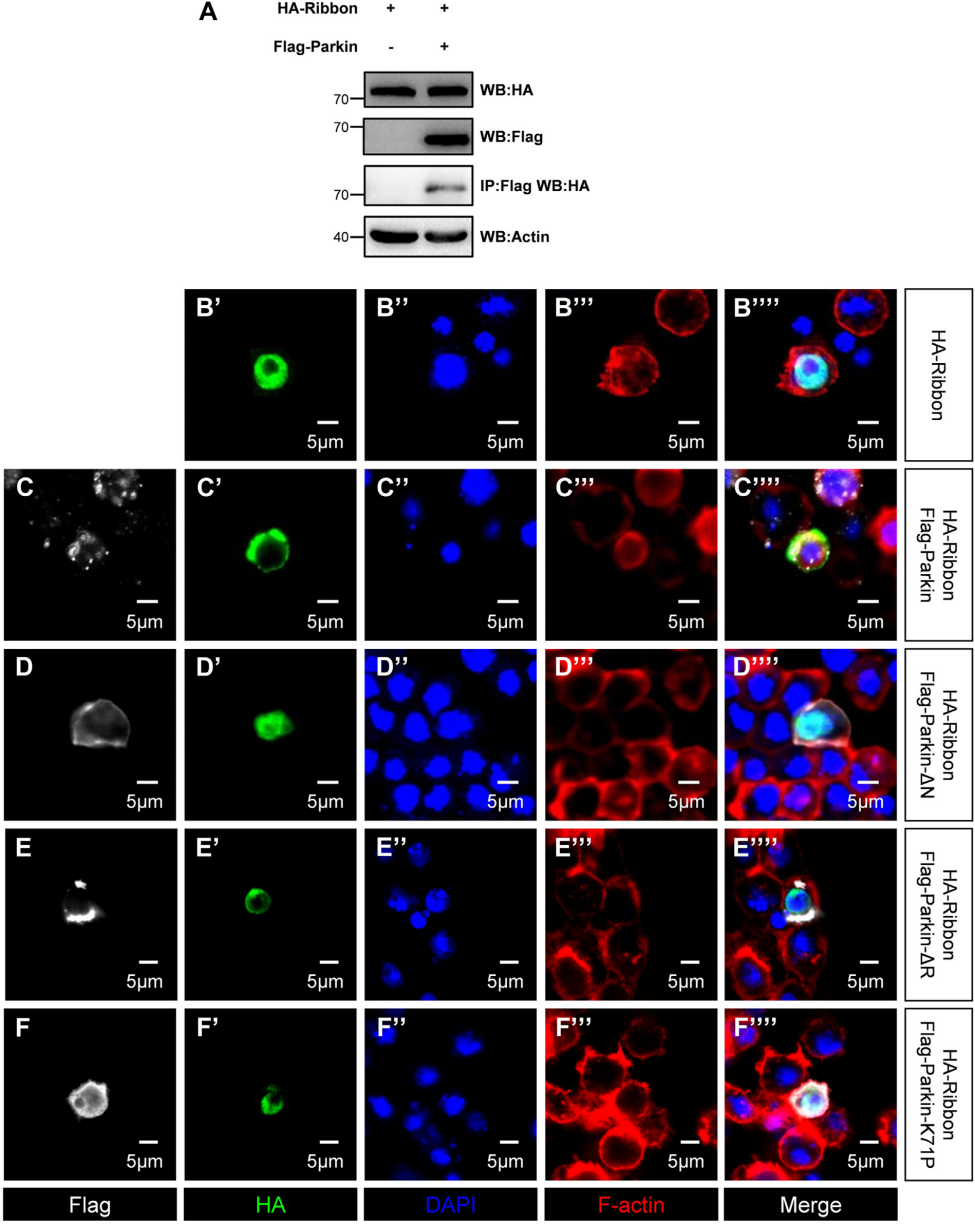
**Parkin modulates Ribbon cellular localization to affect mRpL18 transcription.***A*, Parkin interacted with Ribbon but did not affect its protein level. *B’–B’’’’,* Ribbon alone was localized in the nucleus. *C*–*C’’’’,* co-transfection of *parkin* let Ribbon localize in the cytoplasm. Co-transfected with *parkin-ΔN* (*D–D’’’’*), *parkin-ΔR* (*E–E’’’’*), or *parkin-K71P* (*F–F’’’’*), respectively, Ribbon was localized in the nucleus. For the Ribbon localization analysis, a sample size of n = 20 cells were utilized in *B*–*F* group, respectively. The rates of cells displayed a given phenotype were 0.7, 0.8, 0.9, 0.95 and 0.9 in *B–F* group, respectively.

We next explored how Parkin regulates *mRpL18* transcription through Ribbon. As shown in Figure 5*A*, Parkin bound to Ribbon without affecting its protein level. Given Ribbon as a transcription factor should regulate transcription in the nucleus, we speculated that Parkin may regulate Ribbon cellular localization to affect mRpL18 transcription. In S2 cells, Ribbon alone was localized in the nucleus (Fig. 5, *B*’–*B*’’’’), while co-transfected with Parkin, it was localized in the cytoplasm (Fig. 5, *C*–*C*’’’’), suggesting its cellular localization is regulated by Parkin. To test whether Parkin modulating Ribbon cellular localization is dependent on its E3 ligase activity, we co-transfected HA-Ribbon with Parkin E3 ligase-dead mutants Flag-Parkin-ΔN (Fig. 5, *D*–*D*’’’’), Flag-Parkin-ΔR (Fig. 5, *E*–*E*’’’’) and Flag-Parkin-K71P (Fig. 5, *F*–*F*’’’’), respectively, found Ribbon was localized in the nucleus, suggesting that Parkin prevents Ribbon nuclear transport in its E3 ligase activity dependent manner.

### Parkin prevents Ribbon nuclear import possibly by affecting both Ribbon ubiquitination and Kap-**α**1 stability

**Figure 6:**
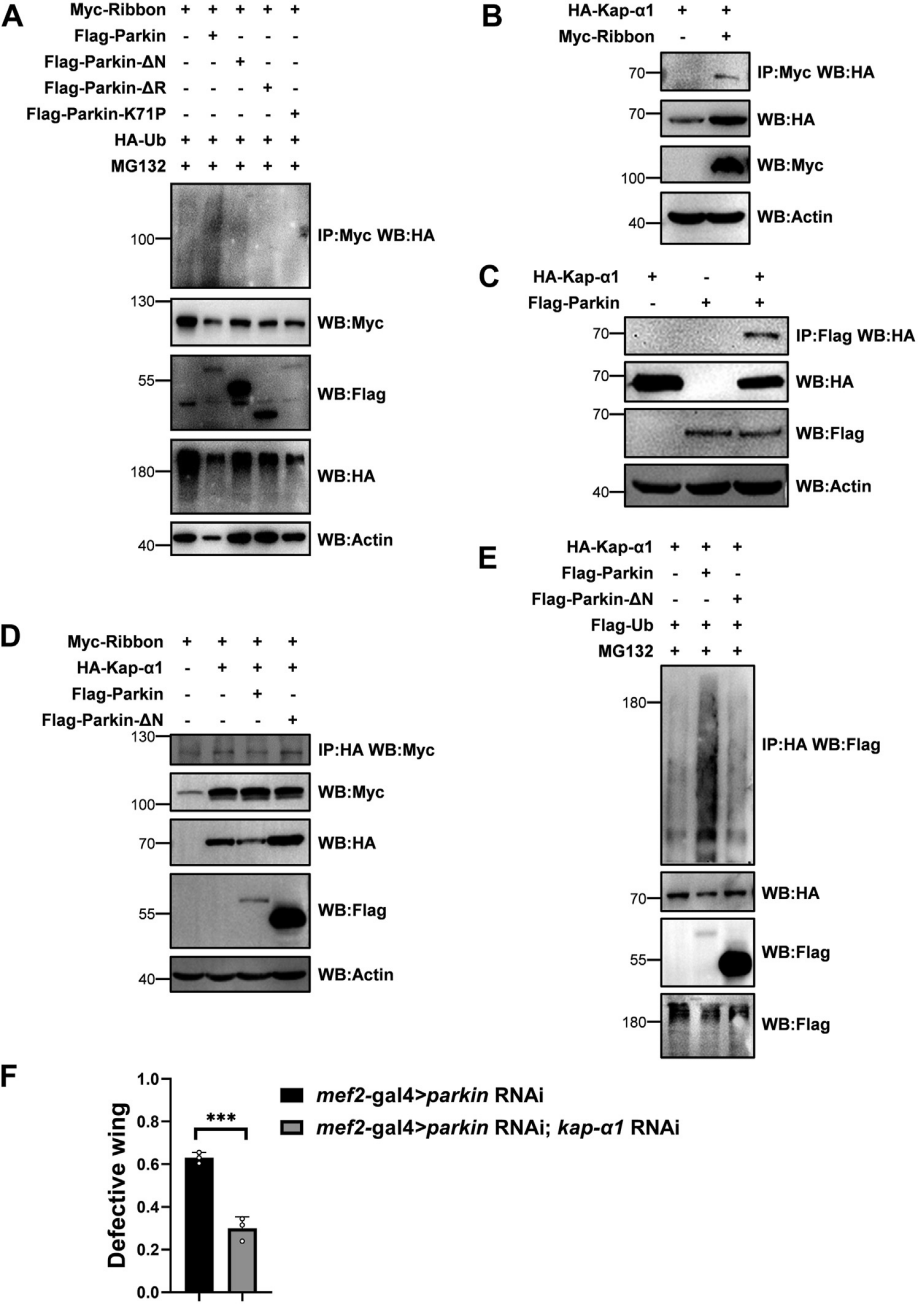
**Parkin prevents Ribbon nuclear import possibly by affecting both Ribbon ubiquitination and Kap-α1 stability.***A*, Parkin modulating Ribbon ubiquitination was dependent on its E3 ligase activity. *B*, Ribbon interacted with importin Kap-α1. *C*, Parkin interacted with Kap-α1. *D*, Parkin could degrade Kap-α1 but did not affect the binding affinity of Ribbon and Kap-α1. *E*, Parkin could mediate the ubiquitination of Kap-α1. *F*, *kap-α1* RNAi partially rescued *parkin* RNAi-mediated defective wing phenotypes. Values in bar graphs represented mean ± SD of three biologically independent experiments. In each biologically independent experiment, n = 200 for the analysis of defective wings. For comparison of two groups, a two-tailed unpaired *t* test was used. ∗ *p*-value < 0.05; ∗∗ *p*-value < 0.01; ∗∗∗ *p*-value < 0.001; ∗∗∗∗ *p*-value < 0.0001.

Since Parkin regulating Ribbon nucleocytoplasmic localization was dependent on its E3 ligase activity, we speculated that Parkin affects Ribbon cellular localization through modulating its ubiquitination. As shown in Figure 6*A*, Parkin did affect Ribbon ubiquitination, supporting that Parkin may mediate Ribbon ubiquitination to restrict its translocation into the nucleus.

Given importins as nuclear transport receptors are responsible for importing cargo proteins into the nucleus through the nuclear pores, we next checked whether Ribbon interacts with them, such as Kap-α1, Impα2, Kap-α3, αKap4, and Impβ (51, 52). As shown in Figures 6*B* and S1, *A*–*D*, Ribbon only interacted with Kap-α1, which also interacted with Parkin (Fig. 6*C*), suggesting that Parkin may regulate Kap-α1 to affect Ribbon nuclear localization. Mechanistically, we speculated that Ribbon entering into the nucleus is dependent on Kap-α1, which Parkin also regulates in its E3 ligase activity-dependent manner. As shown in Figure 6*D*, Parkin did not affect the relative affinity between Kap-α1 and Ribbon; however, Parkin, but not Parkin-ΔN, dramatically downregulated the protein level of Kap-α1, suggesting that Parkin regulates Kap-α1 protein level depending on its E3 ligase activity. Consistently, we found that Parkin but not Parkin-ΔN promoted the ubiquitination of Kap-α1 (Fig. 6*E*), suggesting that Parkin promotes the ubiquitination and degradation of Ribbon receptor Kap-α1 to restrict Ribbon into the nucleus. To further prove this, *kap-α1* was knocked down under the *parkin* RNAi background, we found that *kap-α1* RNAi could partially rescue the *parkin* RNAi-mediated defective wing phenotypes (Fig. 6*F*). Collectively, on the one hand, Parkin may hinder Ribbon nuclear localization by modulating its ubiquitination, leading to the inhibition of its nuclear transport. On the other hand, Parkin may mediate degradation of Kap-α1, leading to a decrease in Ribbon receptor, finally, inhibiting Ribbon into the nucleus. Therefore, Parkin modulates the transcription level of *mRpL18,* possibly by affecting both Ribbon and Kap-α1.

### In addition to modulating mRpL18 transcription, Parkin also affects mRpL18 protein stability

**Figure 7:**
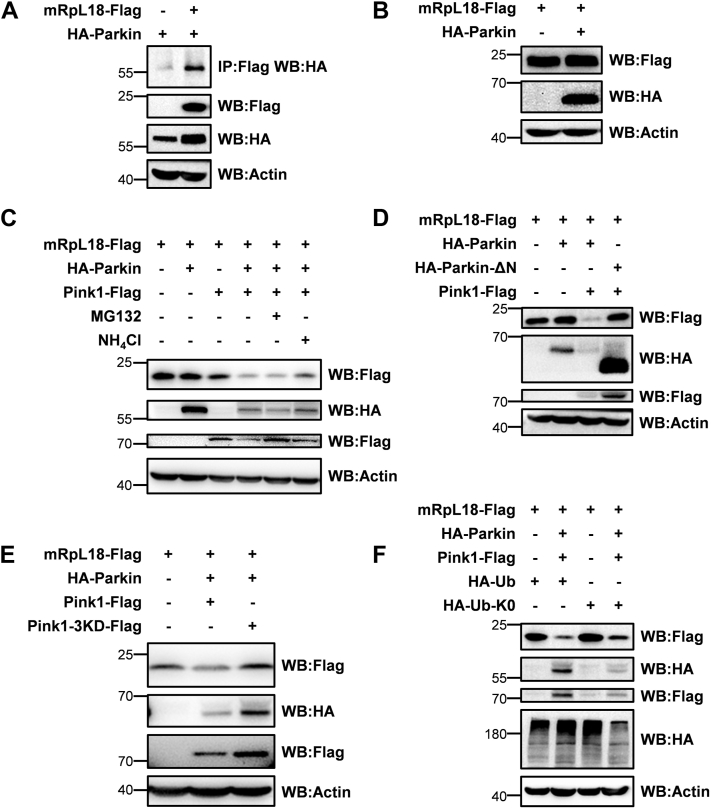
**Parkin affects mRpL18 protein stability.***A*, Parkin bound with mRpL18. *B*, Parkin alone had little effect on mRpL18 protein stability. *C*, Parkin and Pink1 synergistically degraded mRpL18. *D*, Parkin mediating mRpL18 degradation depended on its E3 ligase activity. *E*, Pink1 mediating mRpL18 degradation depended on its kinase activity. *F*, mRpL18 monoubiquitination might be sufficient for Parkin-mediated degradation.

As mentioned previously, Parkin regulated the transcription of *mRpL18*. We next further tested whether Parkin regulates mRpL18 stability. First, we checked whether Parkin interacts with mRpL18. As shown in Figures 7*A* and S2*A*, Parkin interacted with both mRpL18 and mRpL18-Δ(1–33) whose mitochondrial signal peptide had been deleted. When further exploring whether Parkin can degrade mRpL18, we found that Parkin alone had little effect on the protein level of mRpL18 (Fig. 7*B*). Considering Pink1-mediated phosphorylation fully activates Parkin's E3 ligase activity (24, 53, 54), we next added Pink1 to test the effect of Parkin on mRpL18. As shown in Figure 7*C*, we found that Parkin and Pink1 alone had little effects on the protein level of mRpL18, but they together dramatically reduced mRpL18 level, suggesting that Parkin and Pink1 synergistically regulate mRpL18. Of note, the lysosome inhibitor NH_4_Cl but not the proteasome inhibitor MG132 inhibited the degradation of mRpL18, suggesting that it is mainly degraded through lysosomes. In addition, the E3 ligase-dead mutant Parkin-ΔN and the kinase-dead mutant Pink1-3KD no longer regulated mRpL18 (55, 56) (Fig. 7, *D* and *E*), indicating that both Pink1 kinase activity and Parkin E3 ligase activity are necessary for Parkin-mediated mRpL18 degradation.

When mapping which parts of mRpL18 mediate its degradation, we found that mRpL18-Δ(1–33), mRpL18-(34–110), and mRpL18-(111–186) all could be degraded by Parkin (Fig. S2, *B*–*E*), indicating multiple regions of mRpL18 mediate its degradation. Interestingly, when interfering with the polyubiquitination, we found that it did not affect the degradation of mRpL18 (Fig. 7*F*), suggesting that mRpL18 monoubiquitination may be sufficient for Parkin-mediated degradation.

### mRpL18 mediates *parkin* RNAi-triggered mitochondrial hyperfusion by inhibiting mitochondrial fission

As mentioned previously (Fig. 1*E*), overexpression of Drp1, which promotes mitochondrial fission, rescued *parkin* RNAi-mediated phenotypes. Similarly, the overexpression of Fis1, which is the receptor of Drp1, also partially rescued *parkin* RNAi-mediated abnormal wing phenotypes, while overexpression of Marf or Opa1, which promotes mitochondrial fusion (57), caused lethal phenotypes. Overall, these results suggest that *parkin* RNAi-mediated mitochondrial hyperfusion through mRpL18 causes adverse effects.

**Figure 8:**
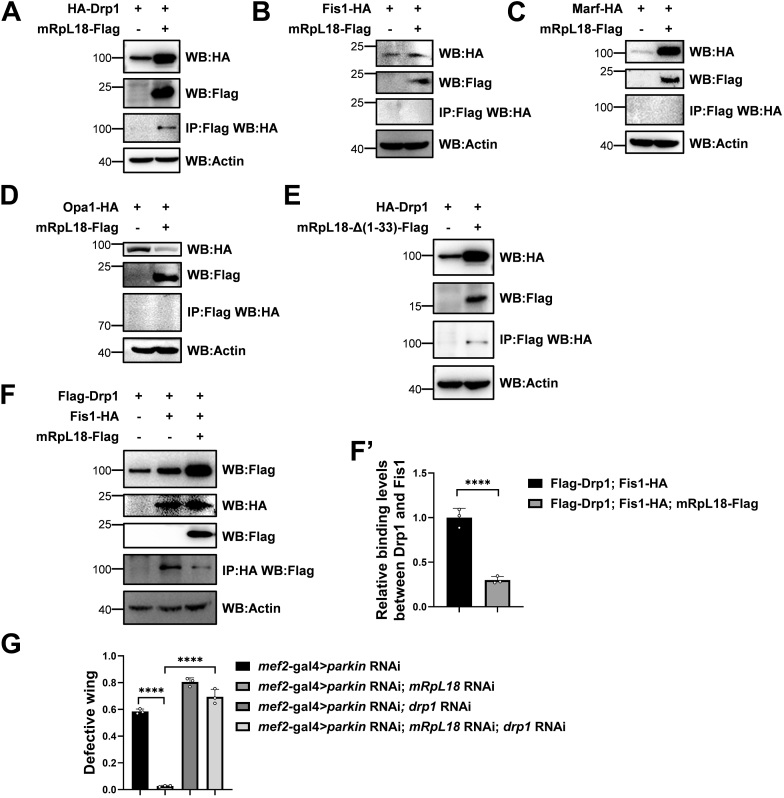
**mRpL18 mediates *parkin* RNAi-triggered mitochondrial hyperfusion by inhibiting mitochondrial fission.***A–D*, mRpL18 bound with Drp1 but not Fis1, Marf and Opa1. *E*, mRpL18-Δ(1–33) bound with Drp1. *F* and *F′*, mRpL18 attenuated the binding between Drp1 and Fis1. Western blots were representative of three biologically independent experiments and the bar graph showed the mean ± SD. *G*, Drp1 functioned at the downstream of mRpL18. Values in (*G*) represented mean ± SD of three biologically independent experiments. In each biologically independent experiment, n = 200 flies. For *F′*, two tailed unpaired *t* test was used. For *G*, one-way ANOVA was used, F (3, 8) = 324.1, *p*-value < 0.0001. The *post hoc* test results following one-way ANOVA were indicated by significant difference star(s), ∗ *p*-value < 0.05; ∗∗ *p*-value < 0.01; ∗∗∗ *p*-value < 0.001; ∗∗∗∗ *p*-value < 0.0001.

When further testing how mRpL18 mediates mitochondrial hyperfusion, we found that mRpL18 bound with Drp1 but not Fis1, Marf, and Opa1 in S2 cells (Fig. 8, *A*–*D*). In addition, Drp1 also interacted with mRpL18-Δ(1–33) (Fig. 8*E*), suggesting that mRpL18 may mediate the *parkin* RNAi phenotypes through interacting with Drp1, leading to tipping the balance of mitochondrial fission and fusion. Fis1, as the receptor of Drp1 on mitochondria, is required for Drp1-mediated mitochondrial fission. Next, we tested whether mRpL18 affects the binding between Drp1 and Fis1. As shown in Figure 8, *F* and *F’*, co-transfection of mRpL18 dramatically weakened the binding between Drp1 and Fis1, suggesting that mRpL18 may competitively bind Drp1 to interfere with the interaction of Drp1 and Fis1, leading to the inhibition of mitochondrial fission. Next, we further tested our hypothesis through *in vivo* genetic experiments. In *parkin* RNAi background, knockdown of *mRpL18* could rescue, while knockdown of *drp1* greatly aggravated the abnormal wing phenotypes. While simultaneous knockdown of *mRpL18* and *drp1* showed similar phenotypes to *drp1* RNAi alone (Fig. 8*G*), indicating that Drp1 functions downstream of mRpL18, *parkin* RNAi phenotypes are mediated by the mRpL18-Drp1 axis.

### Parkin-mRpL18 axis may also apply to the fly dopamine neuron system

**Figure 9:**
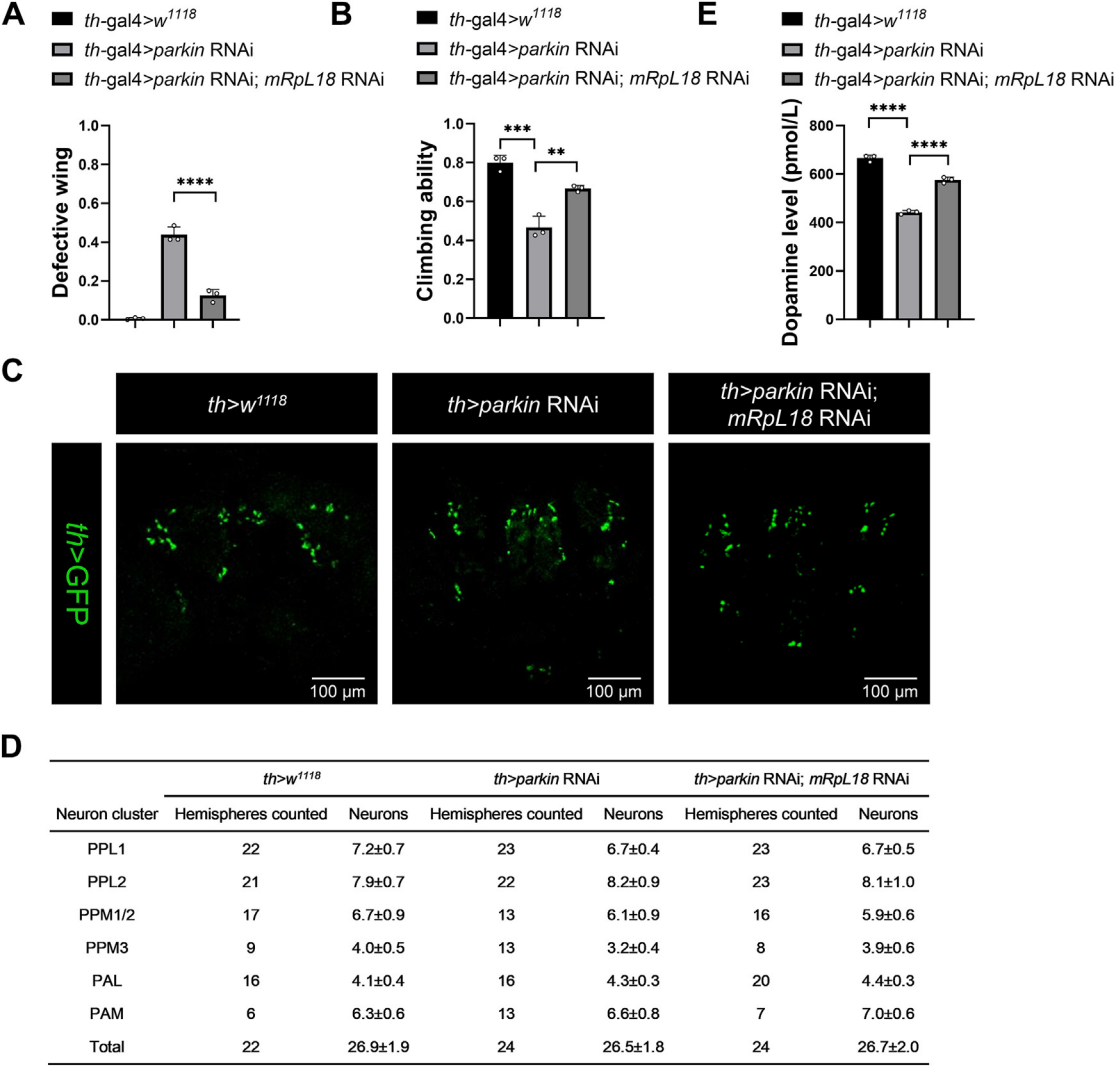
**Parkin-mRpL18 axis may also apply to fly dopamine neuron system.***A*, knockdown of *parkin* in dopamine neurons also produced abnormal wing phenotypes and knockdown of *mRpL18* also rescued the defective wing phenotypes. *B*, knockdown of *parkin* in dopamine neurons reduced the climbing ability and knockdown of *mRpL18* also rescued the defective climbing ability. *C*, there were no changes in the dopamine neuron numbers among WT, *parkin* RNAi and *parkin* RNAi plus *mRpL18* RNAi flies. *D*, average number of *th*>GFP-positive neurons counted in each and total dopamine neuron clusters. *E*, knockdown of *parkin* in dopamine neurons decreased the dopamine level and knockdown of *mRpL18* rescued the dopamine level. Values in bar graphs represented mean ± SD of three biologically independent experiments. In each biologically independent experiment, n = 200 for the analysis of defective wing; n = 100 for the analysis of climbing ability. For the number analysis of dopamine neurons, n was noted in (*D*). Values in table were shown as mean ± SEM. One-way ANOVA was used for (A), (B), and (E), their F and *p*-values were F (2, 6) = 168.7, *p*-value < 0.0001; F (2, 6) = 49.64, *p*-value = 0.0002 and F (2, 6) = 299.0, *p*-value < 0.0001, respectively. The *post hoc* test results following one-way ANOVA were indicated by significant difference star(s), ∗ *p*-value < 0.05; ∗∗ *p*-value < 0.01; ∗∗∗ *p*-value < 0.001; ∗∗∗∗ *p*-value < 0.0001.

Except for the fly muscle system, we further tested whether the Parkin-mRpL18 axis is also applicable to the fly dopamine neuron system. First, we knocked down *parkin* with *th*-gal4 in fly dopamine neurons, found that *parkin* RNAi flies also showed abnormal wing phenotypes and impaired climbing ability; while simultaneous knockdown of *mRpL18* in this background rescued *parkin* RNAi-mediated phenotypes (Fig. 9, *A* and *B*). Next, we checked the number of dopamine neurons in single and total dopamine neuron clusters, found that, similar to Dumitrescu’s result (58), there was no significant difference among WT, *parkin* RNAi, and *parkin* RNAi plus *mRpL18* RNAi flies (Fig. 9, *C* and *D*). When we further examined the dopamine level, we found that dopamine level decreased after *parkin* knockdown, and in this background simultaneous knockdown of *mRpL18* greatly restored dopamine level (Fig. 9*E*), suggesting that despite the total number of dopamine neurons remaining unchanged in *parkin* RNAi flies, there were functional changes, as evidenced by the abnormal wing phenotypes, decreased climbing ability and dopamine level. Importantly, similar to the muscle system, in the *parkin* RNAi background, simultaneous knockdown of *mRpL18* largely rescued the wing phenotypes, climbing ability, and dopamine level in fly dopamine neurons, supporting that the Parkin-mRpL18 axis may also apply to the fly dopamine neuron system.

## Discussion

**Figure 10:**
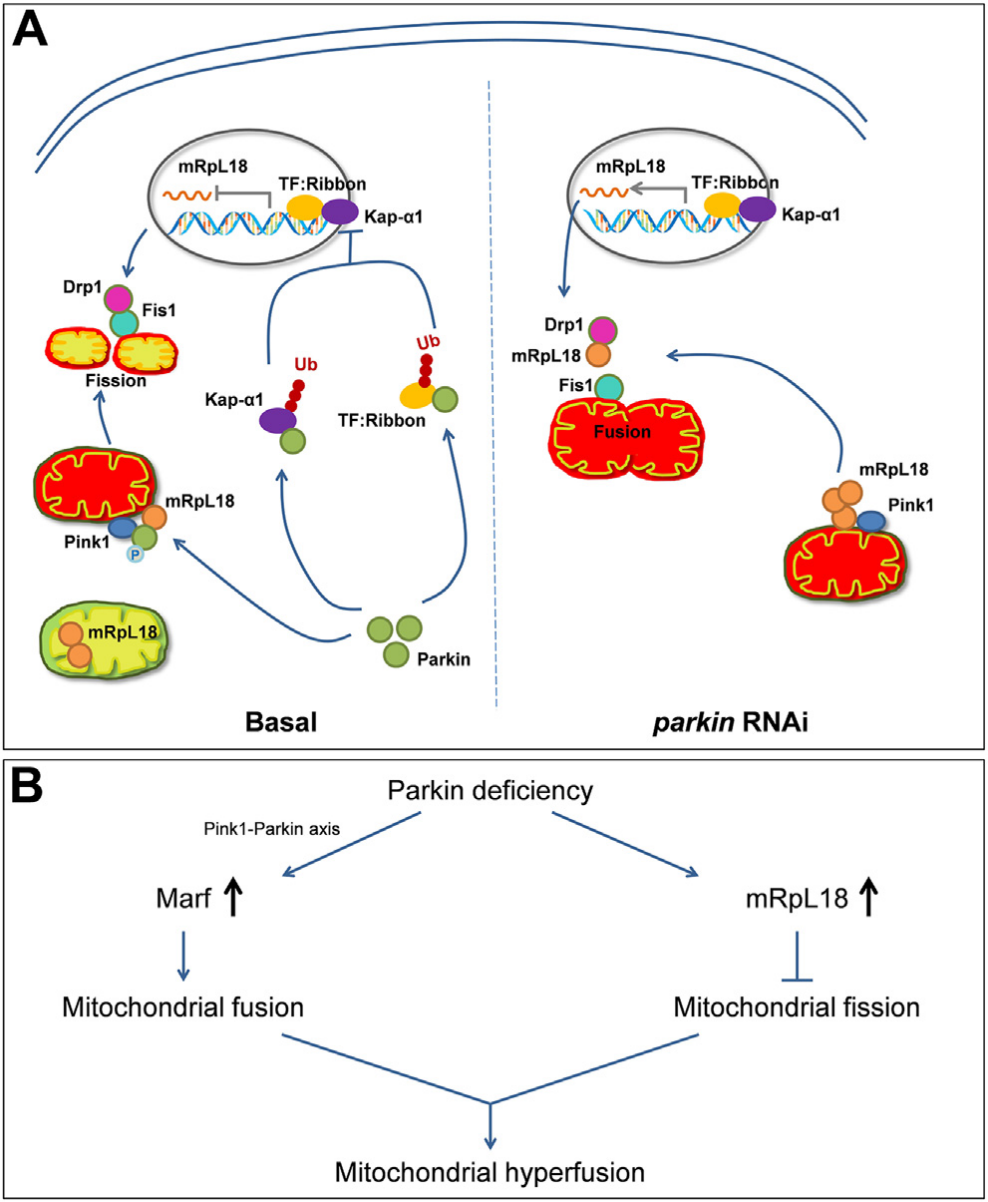
**Working model.***A*, in kinds of mitochondrial defects, Pink1 accumulates on the mitochondrial outer membrane to recruit and activate Parkin which inhibits the mRpL18 transcription and promotes the protein degradation of cytosolic retained mRpL18 in its E3 ligase activity dependent manner. The reduced mRpL18 leads to more Drp1 binding with its receptor, that promotes mitochondrial fission and prevents mitochondrial hyperfusion. However, in the background of *parkin* knockdown, cytosolic accumulation of mRpL18 inhibits mitochondrial fission, that results in mitochondrial hyperfusion and finally impairs mitochondrial function. *B*, loss of *parkin* can cause mitochondrial hyperfusion due to two reasons: (1) Parkin deficiency impairs Pink1-Parkin axis-mediated Marf degradation, which promotes mitochondrial fusion and (2) Parkin deficiency causes mRpL18 accumulation, which impairs Drp1/Fis1-mediated mitochondrial fission. These two ways function together to drive Parkin-mediated mitochondrial hyperfusion phenotype.

Loss of function of *parkin*, one of the causative genes of Parkinson's disease, causes mitochondrial dysfunction, which is closely related to PD. However, the *in vivo* mechanism of how defective Parkin causes mitochondrial dysfunction is far from clear. Surprisingly, until now, a genome-wide RNAi screen to identify the rescue genes of the *parkin* RNAi phenotype has not been conducted. Therefore, we established the mitochondrial defect *Drosophila* model by knockdown of *parkin* and did the unbiased screen, finding that knockdown of *mRpL18* or *CLS* (cardiolipin synthase) rescued the mitochondrial defect phenotypes mediated by *parkin* RNAi. In this paper, we only focused on *mRpL18* and revealed that knockdown of *parkin* upregulated *mRpL18* at both transcription and protein levels in an E3 ligase-dependent manner. Then, more cytosolic mRpL18 decreased the interaction between Drp1 and its receptor Fis1 through competitively binding with Drp1, leading to inhibiting mitochondrial fission and tipping the balance to mitochondrial hyperfusion, finally causing *parkin* RNAi-initiated defects. Our study demonstrates a novel mechanism of how *parkin* RNAi causes mitochondrial dysfunction, indicating that *parkin* RNAi-mediated mitochondrial defects result from changes in mitochondrial morphology by mRpL18. The detailed mechanism is shown in Figure 10*A*. Various mitochondrial defects cause Pink1 accumulation on the mitochondrial outer membrane, which recruits and activates Parkin, leading to inhibiting mRpL18 transcription and promoting cytosolic retained mRpL18 degradation in a Parkin E3 ligase activity-dependent manner. The reduced mRpL18 causes more Drp1 to bind with its receptor, which promotes mitochondrial fission and prevents mitochondrial hyperfusion. However, in the *parkin* knockdown case, cytosolic accumulation of mRpL18 inhibits mitochondrial fission, which results in mitochondrial hyperfusion and finally impairs mitochondrial function.

Of note, a previous study reported that *marf* knockdown could rescue *parkin* mutant fly wing phenotypes (29). Based on this and our results, we thought that impaired *parkin* causes mitochondrial hyperfusion due to two reasons: (1) Parkin defect impairs Pink1-Parkin axis-mediated Marf degradation, which promotes mitochondrial fusion; (2) Impaired Parkin causes mRpL18 accumulation, which inhibits Drp1/Fis1-mediated mitochondrial fission. These two ways function together to drive Parkin-mediated mitochondrial hyperfusion phenotype. Therefore, knockdown of either *marf* or *mRpL18* can prevent mitochondrial hyperfusion, leading to the rescue of *parkin* RNAi-triggered fly wing phenotypes. Overall, our study unveils a new facet of how Parkin regulates the mitochondrial morphology (Fig. 10*B*), suggesting that Parkin mediates the mitochondrial dysfunction through synergy of both Marf and mitochondrial protein mRpL18.

Given that mRpL18, as a mitochondrial ribosomal protein, is necessary for mitochondrial protein synthesis (48, 59, 60, 61), Parkin regulating its transcription and protein levels implies that Parkin may also be involved in the regulation of mitochondrial biosynthesis in addition to regulating mitochondrial morphology. Therefore, Parkin may function as a rheostat that links mitochondrial status to mitochondrial biogenesis. In healthy mitochondria, Parkin is less recruited and activated, leading to more mRpL18 into mitochondria to promote mitochondrial biogenesis. While in mitochondrial defect cases, more Parkin is recruited and activated, leading to a decrease in the level of mRpL18, thus inhibiting mitochondrial biogenesis to prevent waste and blocking mitochondrial hyperfusion. The above hypothesis awaits to be determined in the future.

We have demonstrated that the Parkin-mRpL18 axis is also applicable to the fly dopamine neuron system. Of note, unlike many other mammalian cells, where loss of Parkin function promotes mitochondrial hyperfission (62, 63), in the dopaminergic neurons of rat midbrain, and in the *parkin* patient iPSC-derived dopamine neurons (34, 35), similar to *Drosophila* (29), loss of Parkin function promotes mitochondrial hyperfusion, suggesting that our study may also apply to mammalian dopamine neuron system. Taken together, our study unveils a novel mechanism of how Parkin regulates mitochondrial morphology to affect mitochondrial function, which provides new insights for the understanding and potential treatment of Parkinson's disease.

## Experimental procedures

### *Drosophila* strains and genetics

The RNAi stocks used for genome-wide screens were got from VDRC and NIG. Independent RNAi stocks used were UAS*-parkin* RNAi (VDRC:104363), UAS*-mRpL18* RNAi (NIG:12373R-1), UAS*-ribbon* RNAi (NIG:7230R-2), UAS*-kap-α1* RNAi (NIG:8548R-3), UAS*-drp1* RNAi (VDRC:44156), UAS*-marf* RNAi (NIG:3869R-1, 3869R-2), UAS*-CG4282* RNAi (NIG:4282R-3), UAS*-CG12769* RNAi (NIG:12769R-2), UAS*-CG4854* RNAi (NIG:4854R-2), UAS*-ranshi* RNAi (NIG:9793R-1), and UAS*-chorion factor 2* RNAi (NIG:11924R-2). For experiments involving transgenic flies, constructs were injected into *w*^*1118*^, and multiple independent fly lines were generated and analyzed. Flies were cultured following standard procedures at 25 °C. RNAi experiments were performed at 30 °C except for dopamine neuron numbers and dopamine level experiments at 25 °C.

### Cell culture, transfection, and treatment

Only the S2 cell line was used in this paper. S2 cells were maintained at 25 °C in Schneider’s *Drosophila* medium (S9895, Sigma) supplemented with 10% FBS (F0718, Gibco) and 1% penicillin/streptomycin (Invitrogen). S2 cells were transfected using PEI (Polyethylenimine, 1 μg/μl) with final DNA concentration of 1 μg/ml and final PEI concentration of 3 μg/ml in total culture media. For treatments, S2 cells were treated with MG132 at a final concentration of 10 μM for 4 h and NH_4_Cl at a final concentration of 10 μM for 16 h before harvested.

### Real-time quantitative PCR

For real-time quantitative PCR, total RNA from 10 thoraces of each genotype was isolated using TRIzol (Invitrogen) following standard protocol. The reverse transcription was carried out using PrimeScript RT Reagent Kit with gDNA Eraser (TaKaRa). Real-time quantitative PCR was carried out using SYBR Green JumpStart Taq ReadyMix (Sigma-Aldrich) in a real-time thermal cycler (ABI StepOne Plus). The primers used were as follows: *parkin*, 5′-CATATCGGGGAGTGTCTGCC-3′ (forward), 5′-CGTGGGGGTTCTGCATTTTG-3′ (reverse); *mRpL18*, 5′-CAGGTCGTTCCTACTGGCAC-3′ (forward), 5′-CGTTGCAGGTCATTTCCGTG-3′ (reverse); *actin*, 5′-CGAAGAAGTTGCTGCTCTGGTTGTCG-3′ (forward), and 5′-GGACGTCC CACAATCGATGGGAAG-3′ (reverse).

### Immunoprecipitation and western blot

For immunoprecipitation, cell lysates were incubated with the antibody overnight at 4 °C with end-over-end rotation. The samples were further incubated with protein A/G agarose beads (1:20; Santa Cruz) for 1 h and washed three times with lysis buffer. The beads were resuspended in 2x loading buffer for 5 min at 100 °C before loading onto SDS-PAGE gels and immunoblotting. For western blot, bands were separated by SDS-PAGE using standard procedures and transferred onto a PVDF membrane. The membrane was blocked with 5% non-fat milk in TBST buffer and incubated with primary antibodies overnight at 4 °C. The membrane was washed and incubated in HRP-labeled secondary antibodies for 2 h at room temperature. The bands were visualized using a chemiluminescent detection kit (E411; Vazyme) and captured with a chemiluminescence apparatus (Tanon 5200). The images were analyzed using Image J software. The antibodies used for immunoprecipitation at the following dilutions mouse anti-HA (F7) (1:200; Santa Cruz), mouse anti-Myc (9E10) (1:200; Santa Cruz), and mouse anti-Flag (M2) (1:500; Sigma). The antibodies used for western blot at the following dilutions: mouse anti-ATP5A (1:5000; Abcam); mouse anti-HA (F7) (1:5000; Santa Cruz); mouse anti-Myc (9E10) (1:5000; Santa Cruz); mouse anti-Flag (M2) (1:5000; Sigma); mouse anti-Actin (1:10000, Genscript); mouse anti-Tubulin (1:10000, Abcam); and goat anti-mouse HRP (1:10000).

### Immunofluorescence and confocal microscopy

For analysis of muscles, six thoraces of 4-day-old adult flies for every sample were dissected in relaxing buffer (20 mM PBS PH7.0, 5 mM MgCl_2_, 5 mM EGTA), and then fixed in 4% formaldehyde for 30 min. After washed in PBST (PBS with 0.1% Triton X-100), samples were incubated with the primary antibodies overnight at 4 °C. After washing in PBST, samples were incubated with the fluorescent-labeled secondary antibody for 2 h at room temperature. The analysis procedure of dopamine neurons was adapted from the method of Eduard *et al.* (58). For details, 12 brains of 30-day-old adult flies for every sample were fixed in 4% formaldehyde for 30 min, and then washed with PBST for three times. Thereafter, 60% glycerol was used to mount brain samples. For S2 cell immunostaining, after transfection for 48 h, cells were harvested and washed with PBS. Then cells were fixed in 4% formaldehyde in PBS buffer for 20 min at room temperature, treated with PBST for 20 min and then washed with PBS for three times (20 min each time). The incubation procedure for the antibodies was the same as the above muscle treatment process. Images were captured with Olympus FV1000 confocal microscope. Primary antibodies were used at the following dilutions: mouse anti-ATP5A (1:500; Abcam); mouse anti-HA (F7) (1:200; Santa Cruz); mouse anti-Flag (M2) (1:500; Sigma) and rabbit anti-Flag (1:200; Thermo). Fluorescence probes were used at the following dilutions: Mito tracker (MitoTracker Deep Red FM; 1:1000; Yeasen); F-actin (Rhodamine-phalloidin; 1:200; Sigma) and DAPI (1:1000; Santa Cruz). Fluorescent labeled secondary antibodys used in this study were bought from Jackson ImmunoResearch and were diluted at 1:500.

### Transmission electron microscopy analysis

The transmission electron microscopy analysis was performed according to the described procedure (64, 65). In brief, six thoraces from 4-day-old flies for every sample were fixed at 4 °C overnight in a mixed fixative containing 2% glutaraldehyde and 2% formaldehyde. After rinsing with cacodylate buffer, the samples were fixed in 1% OsO_4_ buffer for 2 h. Then, after rinsing with distilled water, the samples were stained with 2% uranyl acetate for 2 h and rinsed with distilled water. The specimens were dehydrated through a graded series of ethanol and embedded in Epon812. Each ultrathin slice was examined under a transmission electron microscope (Hitachi H-7650).

### ATP assay

For analysis of ATP level, 50 thoraces of 4-day-old adult flies were collected. ATP level was measured with an enhanced ATP assay kit (Beyotime) according to the manufacturer’s protocol. Luminescence was measured on an Infinite M200Pro multifunction reader. The relative ATP level was calculated by dividing the luminescence by total protein concentration, which was determined by the Bradford method.

### Dopamine measurement

On the 30th day after eclosion, 30 flies sustained at 25 °C were collected for every sample, and their heads were separated from their bodies using sharp needles. Thereafter, the heads were homogenized in 300 μl PBS (pH 7.4), and, following this, centrifugation was performed at 12,000*g* for 15 min at 4 °C. Then, dopamine level was measured with an ELISA kit (Bio-Swamp) according to the manufacturer’s protocol (66). The absorbance at 450 nm was measured on a multifunctional ELISA reader (Bio-Tek Synergy H1), and the DA content was presented as pmol/l.

### Climbing assay

For climbing assay, 100 flies of each genotype were randomly selected and then were randomly divided into 10 groups with 10 flies in each group. Each group was transferred to an empty 4-inch glass vial without anesthesia. The glass vial was tapped three times in rapid succession to elicit a negative geotaxis response. After 10 s, measure the number of flies that climbed over the 12 cm-lines in each glass tube. Data from 10 groups of three independent biological experiments for each genotype were analyzed.

### Statistical analysis

Imaging data were analyzed in the program Image J. The data shown in the figures were representative of three or more independent experiments and were tested for normal distributions using Shapiro-Wilk tests. The data shown in the figures were analyzed by Student’s *t* test or one-way ANOVA test. Tukey’s multiple comparisons tests were used for the *post hoc* tests. ∗ *p*-value < 0.05; ∗∗ *p*-value < 0.01; ∗∗∗ *p*-value < 0.001; ∗∗∗∗ *p*-value < 0.0001.

## Data availability

All data that support this study are provided in the article. Data will be shared upon request (contact: zhangqing@nju.edu.cn). No datasets were deposited into a publicly accessible repository.

## Supporting information

This article contains supporting information.

## Conflict of interest

The authors declare that they have no conflicts of interest with the contents of this article.
