# Supplementary material for: Parkin mediates the mitochondrial dysfunction through mRpL18
Source: J Biol Chem. 2025 May 8;301(6):110208. doi: 10.1016/j.jbc.2025.110208 (PMC12163413; doi:10.1016/j.jbc.2025.110208)
Supplement: Supporting information [file mmc1.pdf]

Supporting Information

Supporting information list:

Figure S1. Ribbon does not bind several indicated importins.

Figure S2. Parkin interacts with multiple parts of mRpL18 and mediates their degradation.

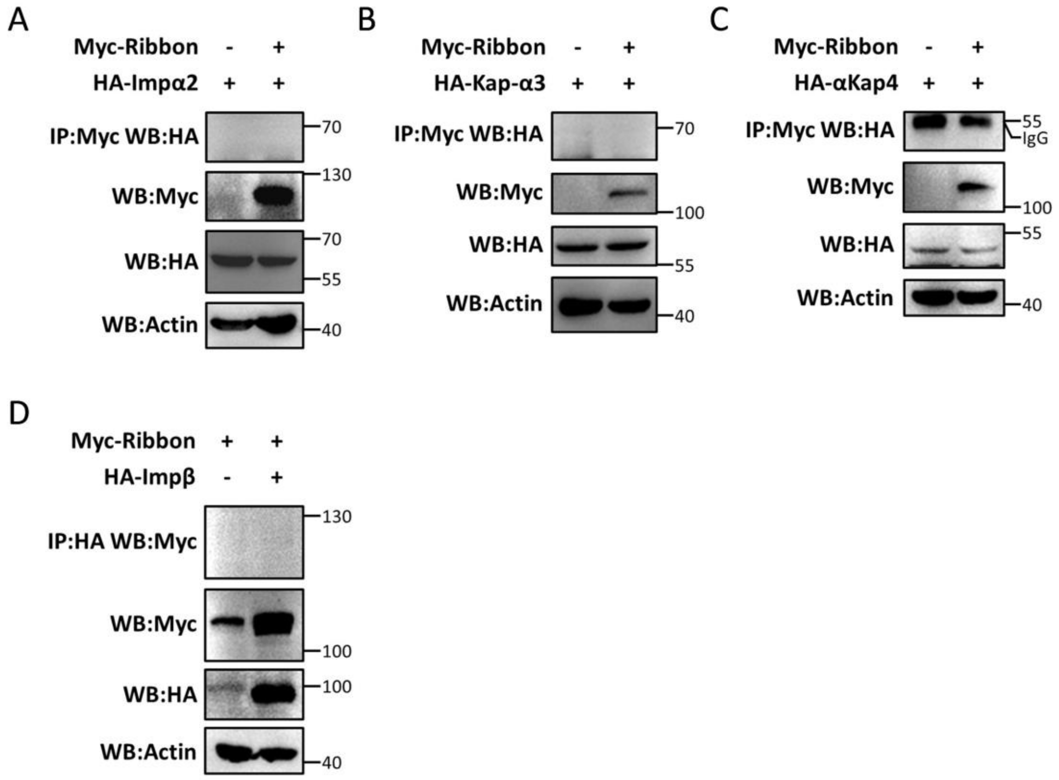

Figure S1. Ribbon does not bind several indicated importins. *A-D,* Ribbon did not bind with importin Impα2 (*A*), Kap-α3 (*B*), αKap4 (*C*) and Impβ (*D*).

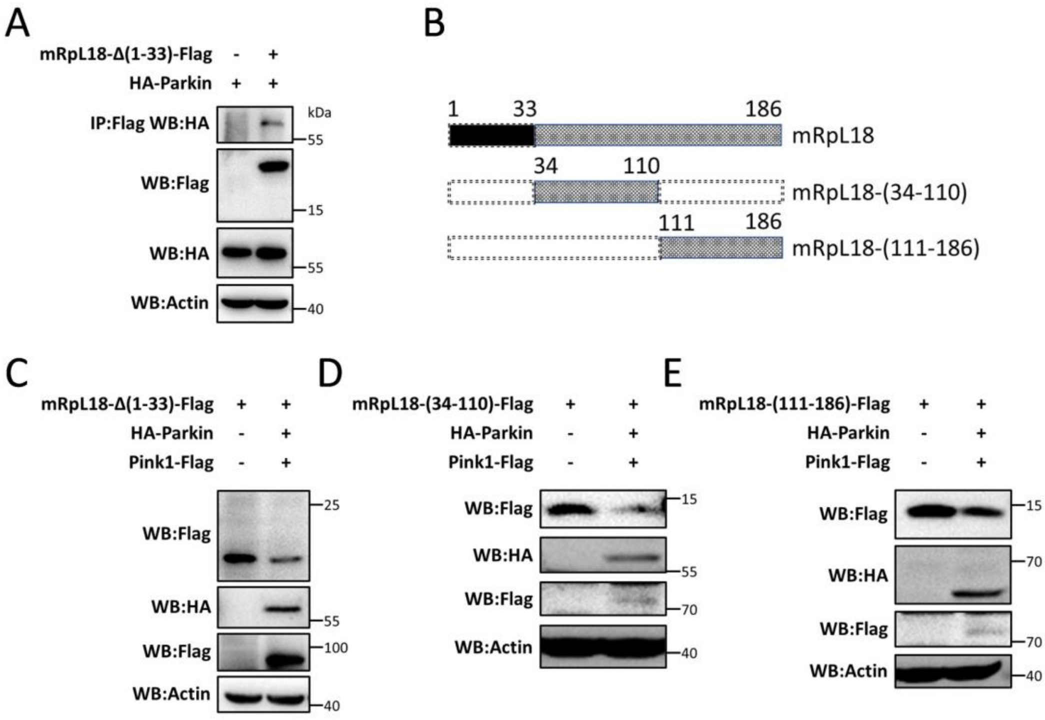

Figure S2. Parkin interacts with multiple parts of mRpL18 and mediates their degradation. *A,* Parkin bound with mRpL18-Δ(1-33). *B,* Schematic diagram of WT and the truncated mRpL18. *C-E,* Different truncated forms of mRpL18 could also be degraded by Parkin.
